# A General Method to Screen Nanobodies for Cytochrome P450 Enzymes from a Yeast Surface Display Library

**DOI:** 10.3390/biomedicines12081863

**Published:** 2024-08-15

**Authors:** Yudong Sun, Cristian Martinez-Ramos, Eugene Chen, Yoichi Osawa, Haoming Zhang

**Affiliations:** 1 Department of Pharmacology, The University of Michigan Medical School, Ann Arbor, MI 48109, USA; yudons@umich.edu (Y.S.); cristiam@umich.edu (C.M.-R.); osawa@umich.edu (Y.O.); 2 Internal Medicine, The University of Michigan Medical School, Ann Arbor, MI 48109, USA; echenum@med.umich.edu

**Keywords:** P450, nanobody, biotinylation, CYP102A1, yeast display library

## Abstract

The availability of yeast surface display nanobody (Nb) libraries offers a convenient way to acquire antigen-specific nanobodies that may be useful for protein structure–function studies and/or therapeutic applications, complementary to the conventional method of acquiring nanobodies through immunization in camelids. In this study, we developed a general approach to select nanobodies for cytochrome P450 enzymes from a highly diverse yeast display library. We tested our method on three P450 enzymes including CYP102A1, neuronal nitric oxide synthase (nNOS), and the complex of CYP2B4:POR, using a novel streamlined approach where biotinylated P450s were bound to fluorescent-labeled streptavidin for Nb screening. The Nb–antigen binders were selectively enriched using magnetic-activated cell sorting (MACS) and fluorescence-activated cell sorting (FACS). After two rounds of MACS, the population of positive binders was enriched by >5-fold compared to the naïve library. The subsequent FACS selection, with a gating of 0.1%, identified 634, 270, and 215 positive binders for CYP102A1, nNOS, and CYP2B4:POR, respectively. The positive binders for CYP102A1 were further triaged based on EC_50_ determined at various antigen concentrations. DNA sequencing of the top 30 binders of CYP102A1 resulted in 26 unique clones, 8 of which were selected for over-expression and characterization. They were found to inhibit CYP102A1-catalyzed oxidation of omeprazole with IC_50_ values in the range of 0.16–2.8 µM. These results validate our approach and may be applied to other protein targets for the effective selection of specific nanobodies.

## 1. Introduction

Nanobodies (Nbs) have seen increasing use in various biomedical research efforts, including structural biology, therapeutic applications, and bio-recognition, due to their small size, stability, and specificity [1,2,3]. Several studies have shown that nanobodies lock flexible proteins or complexes in certain conformations, greatly facilitating structural analysis of protein complexes [4,5,6]. The conventional method of acquiring antigen-specific nanobodies through immunizing llamas is costly and time-consuming [7]. Alternative approaches to acquiring nanobodies through animal-free platforms are gaining popularity. Yeast surface display Nb libraries offer a fast and efficient selection of antigen-specific nanobodies [8,9,10,11]. The yeast surface display Nb library developed by McMahon and coworkers is highly diverse with a naïve library diversity of 5 × 10^8^ [8]. When screened against G-protein-coupled receptors (GPCRs), it yielded nanobodies with affinities ranging from nano- to micromolar. Because of its high diversity, this platform may also be extended to select specific nanobodies for non-GPCR target antigens.

Cytochrome P450s (CYPs or P450s) are a superfamily of heme-containing enzymes involved in the metabolism of xenobiotics [12]. The flexible regions of P450 enzymes play important roles in electron transfer, substrate recognition, and regulation [13,14,15,16]. We envision that the availability of specific nanobodies for P450s would facilitate structure–function studies of these biologically important enzymes. Thus, we have developed a general method for the effective selection of P450-Nb binders from a yeast surface display Nb library. To further facilitate Nb selection, we substituted tight biotin–streptavidin binding for epitope tag–antibody binding, circumventing the use of costly primary and secondary antibodies (Abs) for Nb selection. By biotinylating target antigens and labeling a single cysteinyl variant streptavidin (StvC) with fluorescent probe molecules, positive binders were selected by tight biotin–streptavidin binding using MACS and FACS. We tested our approach on three P450 targets and identified positive binders in each case, which validated the methodology for fast and effective selection of P450-specific nanobodies. This general approach may be extended to other protein targets.

## 2. Materials and Methods

### 2.1. Materials

The yeast surface display Nb library was purchased from Kerafast (Boston, MA, USA). Yeast nitrogen base and Trp dropout media were purchased from US Biological (Salem, MA, USA). Glucose and galactose were acquired from Neogen (Lansing, MI, USA) and BD Bioscience Dickinson (Franklin Lakes, NJ, USA), respectively. Penicillin and streptomycin solution (10,000 units/mL) was acquired from New England Biolabs (Ipswich, MA, USA). LD and LS columns, anti-Cy5/anti-Alexa Fluor 647, and anti-FITC magnetic beads were purchased from Miltanyl Biotec (Gaithersburg, MD, USA). Primary anti-HA rabbit Abs were purchased from Cell Signaling Technology (Danvers, MA, USA) and secondary anti-rabbit IgG conjugated with FITC was purchased from Invitrogen (Carlsbad, CA, USA). Biotinylating reagent Sulfo ChromaLINK biotin was purchased from Vector Laboratories (Newark, CA, USA). Bovine serum albumin (BSA) was acquired from Sigma Aldrich (St. Louis, MO, USA). The StvC plasmid was a generous gift from Dr. Shoji Maeda (The University of Michigan, An Arbor, MI, USA).

### 2.2. Culture Yeast Cell

A frozen stock of yeast from the Nb library was recovered for 24 h at 30 °C/220 rpm in Yglc4.5 media (3.8 g/L-Trp drop-out media supplement, 6.7 g/L yeast nitrogen base, 10.4 g/L sodium citrate, 7.4 g/L citric acid monohydrate, and 20 g/L glucose). At OD_600_ = ~10, the yeast culture was diluted by 100-fold to expand in −Trp/+Glu media (3.8 g/L-Trp drop-out media supplement, 6.7 g/L yeast nitrogen base, and 20 g/L glucose) or to induce Nb expression in −Trp/+Gala media where glucose was replaced with galactose.

### 2.3. Labeling CYP102A1, nNOS, 2B4:POR, and StvC

To facilitate Nb selection, CYP102A1, nNOS, and 2B4:POR were biotinylated, whereas StvC was conjugated with fluorescent probe molecules. Specifically, CYP102A1 and nNOS were biotinylated by reacting with Sulfo ChromaLINK biotin according to the manufacturer’s instructions. Biotin was introduced to the 2B4:POR complex by incorporating the complex in biotinylated amphipols, as described previously [17]. The single cysteinyl residue of StvC was labeled with FITC or Alexa 647 by reacting with either fluorescein-5 maleimide or Alexa Fluor™ 647 C2 maleimide, respectively, according to the manufacturer’s instructions. From this point forward, the StvC protein labeled with FITC or Alexa 647 was referred to as SA-FITC or SA-AF647, respectively.

### 2.4. Selection of P450-Specific Nanobodies by MACS

After the induction of Nb expression in galactose-containing media, a total of ~5 × 10^9^ yeast cells were pelleted and resuspended in Buffer A (20 mM HEPES, pH 7.5, 0.15 M NaCl, 0.1% BSA, 5 mM maltose). To remove false positive binders, aliquots (~20 μL) of SA-AF647 and SA-FITC (10 µM each) and aliquots of anti-AF647 and anti-FITC magnetic beads (300 μL each) were added to 5 mL yeast suspension. The mixture was incubated at 4 °C for one hour. The incubation was then applied to an LD column in a magnetic field to remove non-specific binders. The LD flowthrough fraction was collected, washed, and resuspended in 5 mL of Buffer A, followed by incubation with ~0.5 µM biotinylated antigen for 1 h, then 16 nM SA-FITC for 30 min, and finally, 500 μL anti-FITC magnetic beads for 30 min. The suspension was then applied to an LS column to selectively retain Nb–biotinylated antigen binders in magnetic fields. Yeast cells containing Nb-–biotinylated antigen binders were eluted from the LS column with 10 mL of Buffer A in the absence of magnetic fields. Cell density was determined with a Bio-Rad TC20 cell counter (Bio-Rad, Hercules, CA, USA) before and after the MACS selections to monitor reductions in diversity.

### 2.5. Selection of P450-Specific Nanobodies by FACS

After two rounds of MACS, yeast cells containing biotinylated antigen-specific binders were sorted by two rounds of FACS on a Sony SH800 Cell Sorter (Sony Biotechnology Inc., San Jose, CA, USA). The yeast cells selected by MACS were cultured in −Trp/Glu media and induced in −Trp/+Gala media for 48 h. A total of ~2 × 10^6^ induced yeast cells were pelleted and washed with Buffer A. The cell pellets were resuspended in 200 μL of Buffer A and mixed with 2 μL of primary anti-HA Ab and 0.2 μM biotinylated antigens to monitor the level of Nb expression and antigen–Nb binder, respectively. After being incubated at 4 °C for 40 min, the samples were washed twice and resuspended in 200 μL of Buffer A, to which aliquots of 2 μL anti-rabbit IgG Alexa 488 and 0.1 μM SA-AF647 were added to stain the Nb and Nb–antigen binder, respectively. After being incubated for 40 min, the samples were washed, resuspended in 1 mL of Buffer A, and sorted at a gating of 0.2% in the first round and 0.1% in the second round, together with negative controls and Nb controls. The negative controls and Nb controls were prepared under the same conditions as the samples, except that biotinylated antigens were absent from both controls and the staining agents were also absent from the negative control. Fractions of sorted yeast cells were cultured in −Trp/+Glu media to recover for subsequent studies. To obtain single colonies, recovered yeast cells were plated on YPD plates and incubated at 30 °C.

### 2.6. Triage Nb-CYP102A1 Binders in Yeast Cells

FACS identified 634 yeast cells containing potential specific Nbs for CYP102A1. To select high-affinity nanobodies, these binders were triaged based on their EC_50_s. The EC_50_ values were determined by titrating the yeast cells at increasing concentrations of biotinylated CYP102A1 and monitoring fluorescent intensity from the binders stained with SA-AF467. In a typical experiment, isolated colonies were picked from the YPD plates and cultured at 30 °C/700 rpm in 96well plates containing 0.2 mL of −Trp/+Glu media. In 24 h, the culture was diluted 100fold in −Trp/+Gala media to induce Nb expression and grew at 25 °C/700 rpm for 48 h. A total of 2 × 10^6^ cells were pipetted and resuspended in 0.2 mL of Buffer A. Biotinylated CYP102A1 was then added to final concentrations of 0–0.4 µM and incubated at 22 °C/700 rpm for 30 min. To stain the binders, aliquots of SA-AF647 were used as for FACS experiments. After washing twice with Buffer A, the sample was analyzed by flow cytometry (BD Accuri C6 Plus Flow Cytometer, BD Biosciences, Franklin Lakes NJ, USA). EC_50_s were obtained by fitting fluorescent intensity at various concentrations of biotinylated CYP102A1 using Prism ver 10 (GraphPad Software, LLC, Boston, MA, USA).

### 2.7. Identification and Cloning of Unique Nb Clones of CYP102A1

Based on the triage results, the plasmids of the top 30 binders were prepared with a Zymoprep yeast plasmid miniprep kit (Zymo Research, Irvine, CA, USA) and propagated in bacterial DH5α cells. The Nb gene was confirmed by DNA sequencing at the University of Michigan Sequencing Core Facility, using an oligo primer upstream of the start codon (5′-GCCATGAGATTCCCATCTATC-3′). Unique clones were identified by aligning the DNA sequences of the top 30 binders using MegAlign Pro ver. 14.0 (DNASTAR Inc., Madison, WI, USA). Twenty-six unique Nb genes were cloned to the pET26b vector for expression. The Nb genes were first amplified by PCR to introduce restriction sites of NcoI and XhoI at the 5′- and 3′-terminius, respectively. The gene fragment flanked by the two restriction sites was inserted into the pET26b vector to construct plasmid pET26b-Nb-6×His. It is of note that the construct contained a His_6_ tag on the C-terminus to facilitate purification.

### 2.8. Over-Expression and Purification of Selected Nanobodies of CYP102A1

Nanobodies were over-expressed in bacterial Rosetta 2 cells, as previously described [8]. After harvest, the cell pellets were washed with 1×PBS buffer and resuspended in 100 mL of Buffer B (0.2 M Tris-HCl, pH 8, 0.5 M sucrose, 0.5 mM EDTA). After being cooled on ice, the suspension was subjected to osmotic shock by adding 200 mL of ice-cold deionized water. After centrifugation at 20,000× *g* for 20 min, the supernatant was recovered and loaded onto a PROTEINDEX Ni-Penta column for purification, as described [14]. After being eluted from the Ni-Penta column, Nb-containing fractions were concentrated and dialyzed in 1 L of Buffer B (20 mM HEPES, pH 7.5, 0.1 M NaCl, 15% glycerol). The concentration of nanobodies was determined using a Bio-Rad protein assay, based on calibration with BSA. Purified nanobodies were aliquoted and stored at −80 °C until use.

### 2.9. Characterization of CYP102A1-Specific Nanobodies

The oligomeric state of purified nanobodies was analyzed using size-exclusion chromatography (SEC), as we previously reported [14]. Aliquots of purified Nb solution were loaded onto a Superdex 200 Increase SEC column (10 × 300 mm, Cytiva Life Sciences, Marlborough, MA, USA) and eluted at a flow rate of 0.7 mL/min in 20 mM HEPES buffer (pH 7.4) containing 0.1 M NaCl and 0.01% sodium azide. To examine the effects of nanobodies on the catalytic activity of CYP102A1, we determined the activity for omeprazole hydroxylation, as described previously [18]. The IC_50_ values for the inhibition of omeprazole hydroxylation were determined at various concentrations of nanobodies (0–5 µM). The IC_50_ values were obtained by fitting the dose–response curves using GraphPad Prism ver 10 (GraphPad software LLC, Boston, MA, USA).

### 2.10. Over-Expression and Purification of P450s, POR, and StvC in E coli

Variant A82F CYP102A1 was used in this study and was over-expressed and purified, as described previously [14]. Unlike wild-type CYP102A1, this variant is capable of oxidizing small-molecule substrates like omeprazole [19]. CYP2B4, nNOS, and POR were over-expressed and purified, as previously reported [20,21]. StvC was over-expressed in E coli BL21(DE3) cells and purified by refolding the StvC inclusion bodies, as described previously [22].

## 3. Results and Discussions

### 3.1. Screening Workflow and Strategy

**Figure 1:**
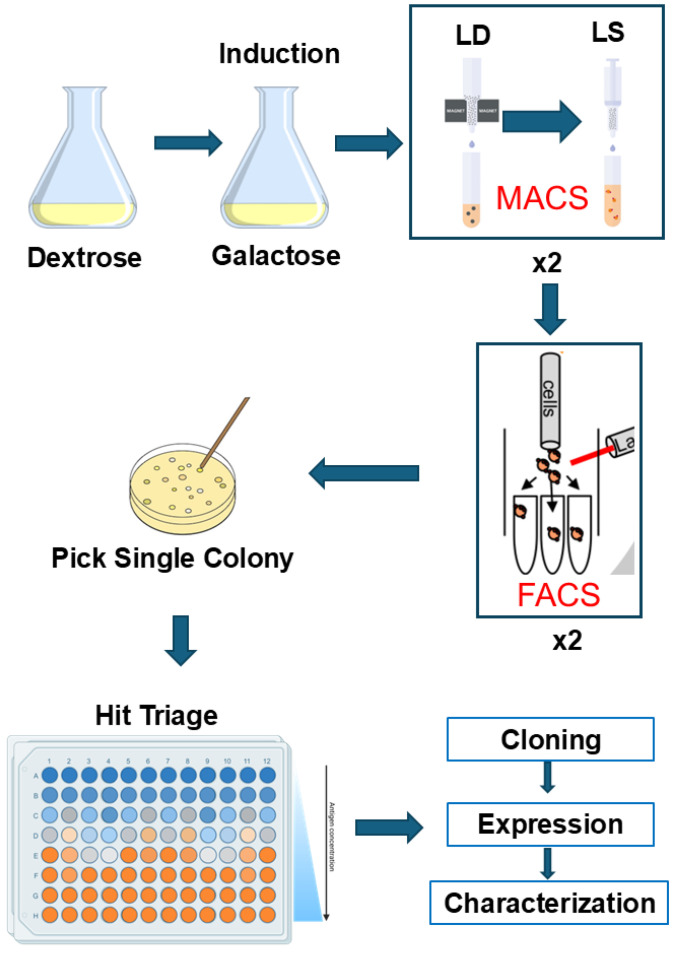
Overview of workflow and Nb selection strategy. Yeast cells were cultured in glucose-containing media and transferred to galactose-containing media to induce Nb expression. Two rounds of MACS and FACS were performed to enrich Nb–antigen binders. The binders were triaged based on EC_50_s, followed by functional characterization. LD, depletion column to remove false positive binders; LS, selection column to select positive binders.

Efficient selection of top Nb–antigen binders depends on screening workflow and strategy. Figure 1 illustrates an overview of our workflow and strategy. After induction of Nb expression, two rounds of MACS were performed to reduce cell diversity, followed by two rounds of FACS to select top binders and reduce false positives. In addition, we introduced a hit triage step after two rounds of FACS to select high-affinity binders. This triage step was made feasible because we substituted tight biotin–streptavidin binding in Nb selection for conventional selection by primary and secondary Abs. The use of relatively large quantities of primary and secondary Abs in the hit triage step would be prohibitively costly.

We utilized two different methods to introduce biotin to antigens as shown in Figure S1 (see the Supplementary Materials). The first method involves the biotinylation of soluble CYP102A1 and nNOS with a labeling reagent, Sulfo ChromaLINK biotin. This reagent reacts with lysine residues of antigens to introduce biotin and contains a UV-traceable chromophore for monitoring labeling reactions and quantifying the number of biotin molecules attached to antigens. On average, approximately four to six biotin molecules were attached to CYP102A1 and nNOS. The second method involves the use of biotinylated amphipol A_8–35_ to incorporate the complex of 2B4:POR. As we reported previously [17], four amphipol molecules formed a nanoparticle into which the 2B4:POR complex is embedded. Since each biotinylated amphipol contained approximately one biotin [23], the formation of the complex of 2B4:POR in biotinylated amphipol introduced four biotin molecules. To introduce fluorescent probes for Nb detection, StvC was conjugated with either FITC or Alexa 467. Tight binding between biotinylated antigens and fluorescent-labeled StvC affords direct detection of Nb–antigen binders.

### 3.2. MACS and FACS Selection of Nb–Antigen Binders

**Figure 2:**
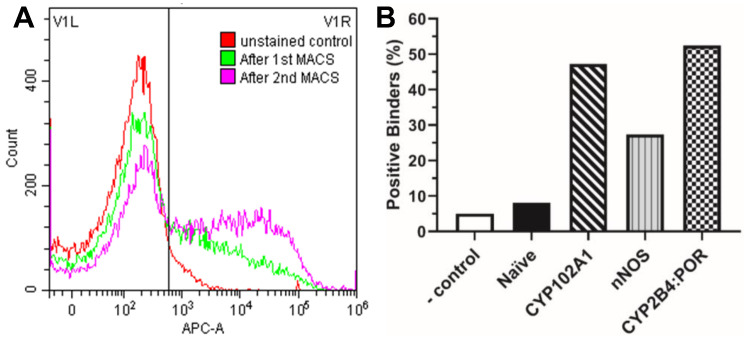
Enrichment of positive binders by MACS. (**A**) Representative results from flow cytometry analysis of yeast cells after the 1st (green) and 2nd MACS (purple), in comparison with unstained control (red). The vertical line indicates gating at 6 × 10^2^ in APC-A channel reading SA-AF647. (**B**) Percent of positive binders of the three antigens based on flow cytometry analysis after the 2nd MACS, compared to the unstained control. Naïve library refers to the natural diversity without prior selection for target antigens.

The introduction of tight biotin–streptavidin binding resulted in the efficient selection of Nb–antigen binders by MACS and FACS. As shown in Table 1, selection by MACS effectively reduced cell diversity for all three antigens. In the first round of MACS, the cell diversity decreased to <1% in all three cases, eliminating most non-binders. In the second round of MACS, the cell diversity was observed at 10, 17, and 8.4% for CYP102A1, nNOS, and CYP2B4:POR, respectively, increasing by >15-fold compared with those in the first round. This is consistent with the increasing population of positive binders revealed by flow cytometry. As shown in Figure 2A, the unstained yeast cells (negative controls) showed basal fluorescent intensity from SA-AF647. In marked contrast, a significant increase in cell population was observed with the high-intensity (>6 × 10^2^) region following MACS selection, with a concomitant decrease in the low-intensity region. This suggests that MACS selection enriched positive binders, which is reinforced by a further increase in the population of positive binders after the second round of MACS selection. The percentage of positive binders for the three antigens after the second round of MACS selection is presented in Figure 2B, in comparison with the unstained control and naïve library. As shown, the positive binders for CYP102A1, nNOS, and 2B4:POR were enriched to 47.2, 27.4, and 52.4% after two rounds of MACS, substantially higher than 8.1% for the naïve yeast cells, indicative of the effective selection by MACS.

**Figure 3:**
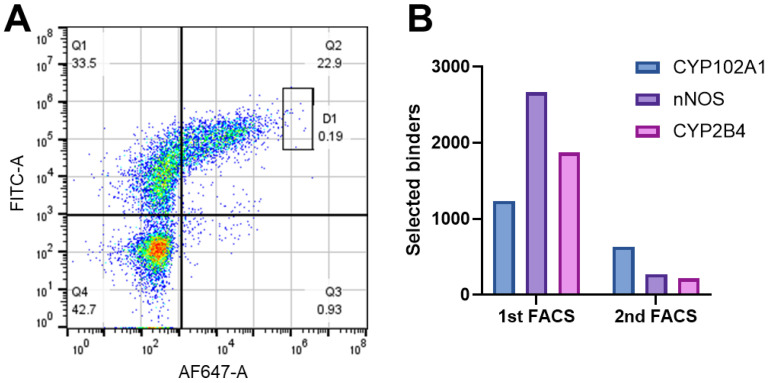
Selection of positive binders by FACS. (**A**) Flow cytometry analysis of Nb-CYP102A1 binders. FITC-A intensity reflects levels of Nb expression, whereas AF647-A represents Nb-CYP102A1 binders. Top binders appear in the Q2 quadrant. (**B**) The number of positive binders selected from two consecutive rounds of FACS for CYP102A1, nNOS, and 2B4:POR.

To further narrow down the top binders, two consecutive rounds of FACS were performed and the results are presented in Figure 3. It is of note that the surface-displayed Nb contains a C-terminal HA-tag, enabling its detection in flow cytometry analysis with primary anti-HA Ab and secondary IgG Ab conjugated with FITC. As shown in Figure 3A, top binders appeared in the boxed region gated at 0.2% in the first FACS, yielding 1263, 2666, and 1870 binders for CYP102A1, nNOS, and 2B4:POR, respectively. The number of binders was reduced to 634, 270, and 215 in the second round with a more stringent gate of 0.1%, as shown in Figure 3B. It is clear that cell sorting by FACS is highly effective in selecting top binders.

### 3.3. Triage Top Binders of CYP102A1

**Figure 4:**
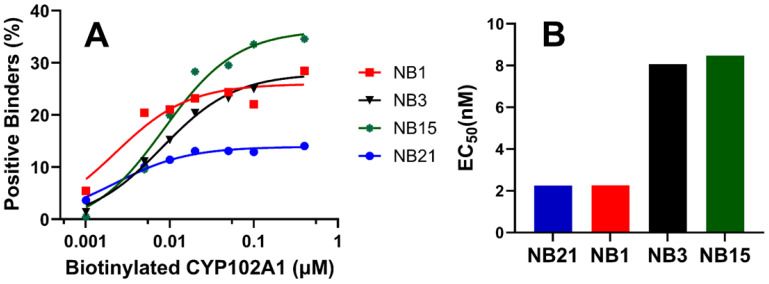
Triage of the positive binders of CYP102A1 based on their EC_50_ values. (**A**). Representative dose–response curves of four of the selected Nb-CYP102A1 binders. The X-axis is displayed on a log scale. (**B**). EC_50_ values were determined from the dose–response curves using GraphPad Prism (ver. 10).

Two rounds of FACS identified 634 binders for CYP102A1. This relatively large number of positive binders necessitated a triage process for subsequent characterization. Given that we utilized tight biotin–streptavidin binding for Nb detection, we could afford to rank these binders based on their relative binding affinities, EC_50_s. The results of four representative binders are shown in Figure 4. As shown in Figure 4A, the signal from SA-AF647-biotin moiety of Nb-CYP102A1 binders, which is proportional to the population of positive binders, exhibited typical dose responses. This dose–response curve allowed us to determine the EC_50_s to be 2.2, 2.3, 8.1, and 8.5 nM for the four nanobodies, namely Nb21, Nb1, Nb3, and Nb15, respectively (Figure 4B). The EC_50_s of the top 30 binders from this triage step are provided in Figure S2 (see the Supplementary Materials), all of which display dose responses resulting in EC_50_s in the range of 2.2–8.5 nM.

### 3.4. Characterization of CYP102A1-Specific Nanobodies

**Figure 5:**
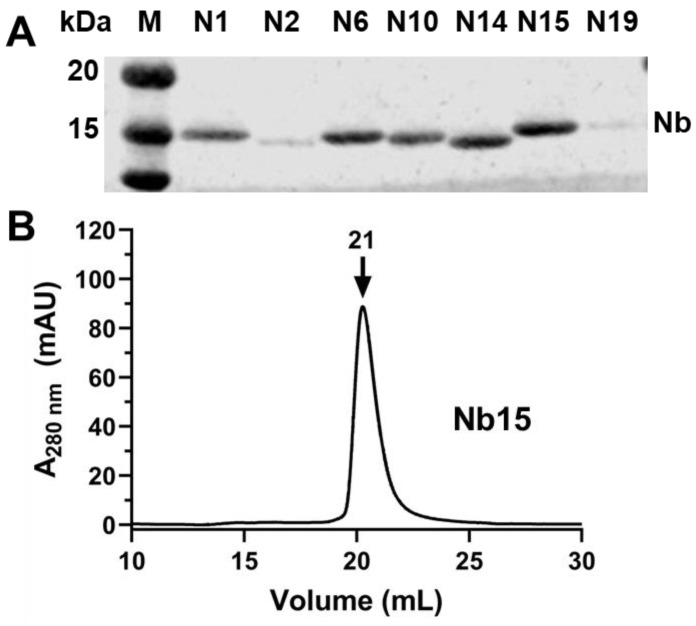
Characterization of representative nanobodies by SDS-PAGE and SEC. (**A**) SDS-PAGE analysis of seven purified nanobodies. Legend: M, protein marker; N1, N2, N6, N10, N14, N15 and N15 represent corresponding nanobodies. (**B**) SEC elution profile of Nb15 on a Superdex 200 Increase SEC column. See Methods and Materials for more details.

Based on the results from hit triage, we selected the top thirty candidates of CYP102A1-specific nanobodies for subsequent characterization. DNA sequencing results confirmed 26 unique nanobodies with highly variable complementarity-determining regions (CDRs). Most of the sequence variability is located in CDR3, as shown in Figure S3 (see the Supplementary Materials). This region is mainly responsible for antigen recognition, as previously reported [8]. As expected, purified nanobodies migrated at ~15 kDa as revealed by SDS-PAGE (see Figure 5A), consistent with their molecular weight. The behavior of purified nanobodies in size-exclusion chromatography varied significantly among them, some of which showed mono-dispersive elution, as shown in Figure 5B, whereas others showed elution of various oligomers. We selected nanobodies with mono-dispersive elution only for functional characterization.

**Figure 6:**
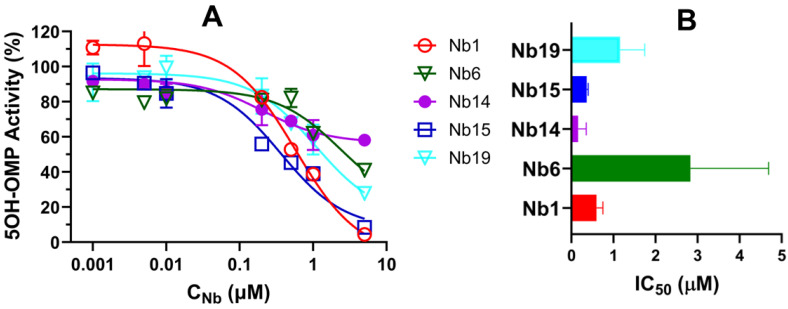
Inhibition of 5-hydroxy omeprazole (5OH-OMP) activity of CYP102A1 by selected nanobodies. (**A**) Dose–response curves for inhibition of CYP102A1 activity. The X-axis is displayed on a log scale. The relative 5OH-OMP activity was normalized to the control containing no nanobodies. (**B**) Calculated IC_50_s from the dose–response curve by fitting the curves with GraphPad Prism ver 10. The experiments were performed in duplicate, as described in Materials and Methods.

The effect of nanobodies on the catalytic activity of CYP102A1 was evaluated by determining the oxidation of omeprazole (OMP) to its 5-hydroxy metabolite 5OH-OMP in the presence of increasing concentrations of nanobodies. The results are shown in Figure 6. It is of note that CYP102A1 functions as a homodimer and requires significant conformational changes to facilitate electron transfer. We postulated that recognition of flexible regions of CYP102A1 may inhibit its catalytic activity. As shown in Figure 6A, the 5OH-OMP activity was progressively inhibited by increasing concentrations of nanobodies, exhibiting a typical dose–response curve. The IC_50_s were determined to be 0.60 ± 0.15, 2.8 ± 1.8, 0.16 ± 0.11, 0.37 ± 0.031, and 1.2 ± 0.58 µM for Nb1, Nb6, Nb14, Nb15 and Nb19, respectively. These nanobodies, capable of inhibiting CYP102A1 with sub-micromolar affinity, could be useful tools to probe the structure–function of CYP102A1, especially for understanding the roles of flexible regions in P450 catalysis.

## 4. Conclusions

We developed a general method to screen for P450-specific nanobodies from a yeast display library. The method was tested with soluble CYP102A1 and nNOS, as well as the complex of membrane-bound 2B4:POR. Substitution of tight biotin–streptavidin binding for conventional primary and secondary antibodies affords two advantages: (1) It reduces the screening cost so that hit triage involving a relatively large volume can be performed to facilitate selection. (2) Membrane protein or protein complexes can be incorporated in biotinylated amphipol without further modification of protein. In the case of CYP102A1, we identified specific nanobodies that inhibit the catalytic activity with sub-micromolar affinity that may be useful for investigating the structure and function of P450 catalysis.

## Figures and Tables

**Table 1:** Reduction in yeast cell diversity for CYP102A1, nNOS, and the 2B4:POR complex after each round of MACS. Diversity is referred to as the ratio of the total number of yeast cells after MACS to that of before MACS.

MACS	CYP102A1		nNOS		2B4:POR	
	Round 1	Round 2	Round 1	Round 2	Round 1	Round 2
Before	4.1 × 10^9^	3.0 × 10^9^	3.0 × 10^9^	1.8 × 10^9^	3.4 × 10^9^	2.5 × 10^9^
After	2.7 × 10^7^	3.0 × 10^8^	1.0 × 10^7^	3.2 × 10^7^	1.6 × 10^7^	2.1 × 10^8^
Diversity	0.66%	10%	0.33%	17%	0.47%	8.4%

## Data Availability

Data are available upon request from the authors due to intellectual property.

## Supplementary Materials

The following supporting information can be downloaded at https://www.mdpi.com/article/10.3390/biomedicines12081863/s1: Figure S1: Illustration of biotin–streptavidin binding used for nanobody detection; Figure S2: EC_50_s of top 30 positive binders of CYP102A1; Figure S3: Sequence alignment of top 26 unique nanobodies for CYP102A1.
