# Supplementary material for: A General Method to Screen Nanobodies for Cytochrome P450 Enzymes from a Yeast Surface Display Library
Source: Biomedicines. 2024 Aug 15;12(8):1863. doi: 10.3390/biomedicines12081863 (PMC11351928; doi:10.3390/biomedicines12081863)
Supplement: Supplementary file 1 [file biomedicines-12-01863-s001.zip › biomedicines-3152309-supplementary.pdf]

Supplementary Materials

A General Method to Screen Nanobodies for Cytochrome P450 enzymes from a Yeast Surface Display Library

Yudong Sun[1], Cristian Martinez-Ramos[1], Eugene Chen[2], Yoichi Osawa[1], and Haoming Zhang[1*]

[1]Department of Pharmacology and [2]Internal Medicine, The University of Michigan Medical School, Ann Arbor, MI 48109

Table of Contents

Figure S1. Illustration of biotin-streptavidin binding used for nanobody detection.

Figure S2. EC_{50}s of top 30 positive binders of CYP102A1

Figure S3. Sequence alignment of top 26 unique nanobodies for CYP102A1.

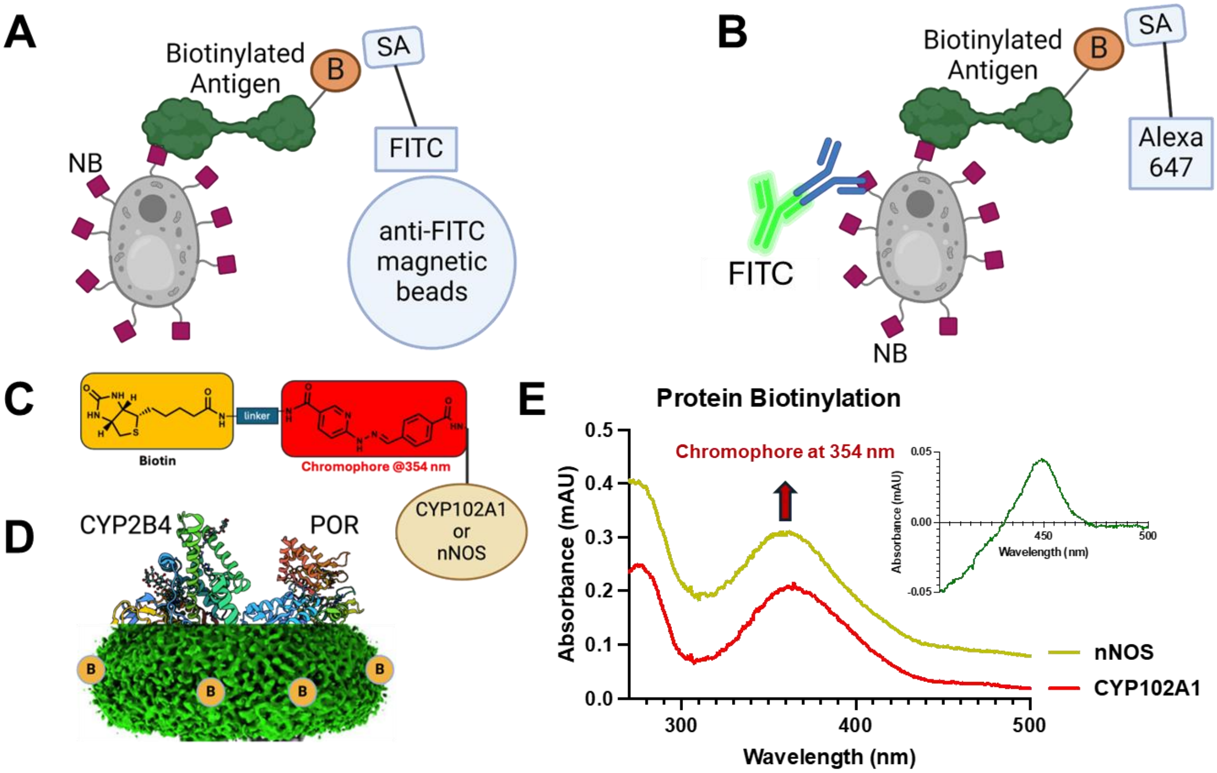

Figure S1. Illustration of Biotin-streptavidin binding used for nanobody detection in MACS and FACS. A) Detection scheme used in MACS. Expressed Nb is attached to the surface of yeast cells and recognizes biotinylated antigen which in turn tightly binds to streptavidin labeled with FITC. B). Detection scheme used in FACS. The Nb-antigen binders is detected by monitoring the fluorescent intensity from Alexa 647 whereas expression of nanobody is detected using conventional primary and secondary Ab. C). Biotinylating reagent used to attach biotin to CYP102A1 and nNOS. (D). Incorporation of the complex of 2B4:POR in biotinylated amphipol. The green bell represents amphipol nanoparticles. E). Absorption spectra of biotinylated CYP102A1 and nNOS. The absorption peak at 350 nm is originated from UV-traceable chromophore from Sulfo ChromaLINK biotin. Inset: ferrous carbon monoxide spectrum of biotinylated CYP102A1. The absorption peak at 450 nm indicates the intactness of the active site. Legend: Nb, surface displayed nanobody; B, biotin; SA, streptavidin.

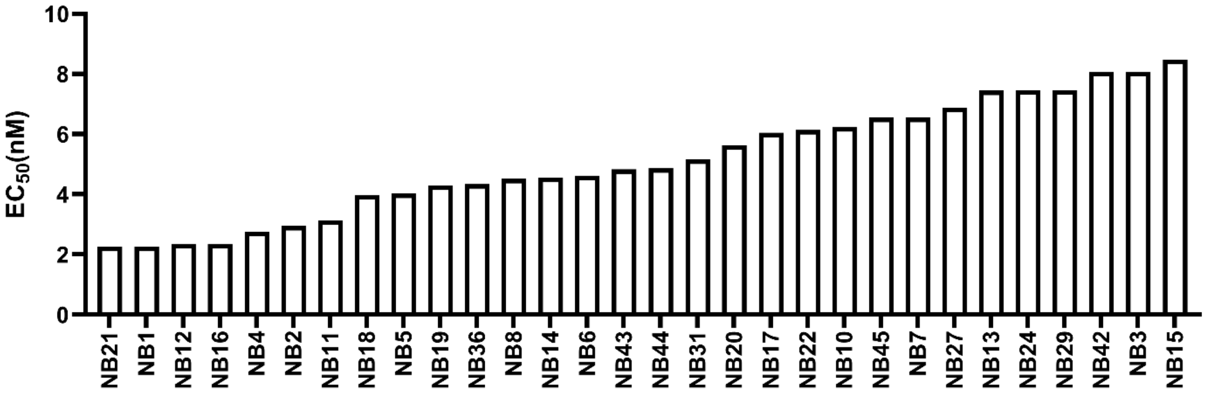

Figure S2. Ranking the top 30 positive binders for CYP102A1 from the hit triage step based on their EC_{50}s. Yeast cells were titrated with increasing concentrations of biotinylated CYP102A1 to obtain a dose response curve from which EC_{50}s were determined by fitting the curve using GraphPad Prism as described in Methods and Materials.

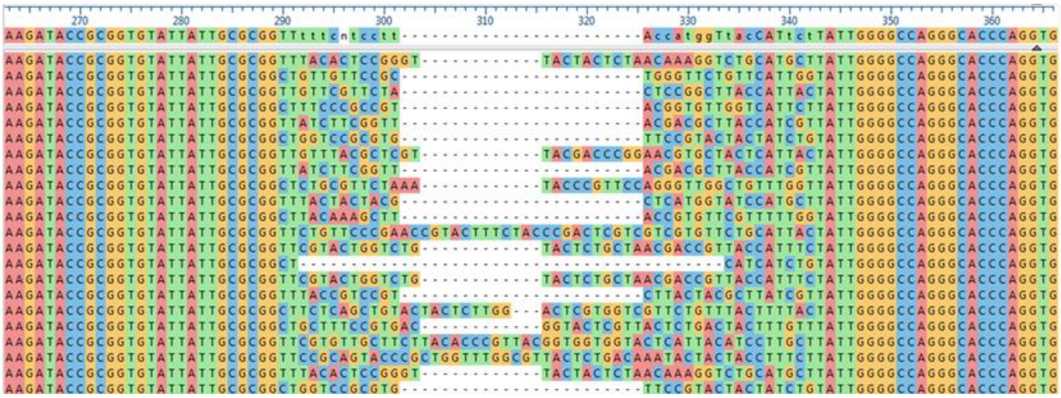

Figure S3. Alignment of unique Nb sequences in the CDR3 region identified from the hit triage step for CYP102A1.
